# Prognostic Significance of Activated Monocytes in Patients with ST-Elevation Myocardial Infarction

**DOI:** 10.3390/ijms241411342

**Published:** 2023-07-12

**Authors:** Mohamed Abo-Aly, Elica Shokri, Lakshman Chelvarajan, Wadea M. Tarhuni, Himi Tripathi, Ahmed Abdel-Latif

**Affiliations:** 1 Gill Heart and Vascular Institute, University of Kentucky, Lexington, KY 40536, USA; 2 Cardiovascular Division, Department of Medicine, Perelman School of Medicine, University of Pennsylvania, Philadelphia, PA 19104, USA; 3 Canadian Cardiac Research Center, Department of Internal Medicine, Division of Cardiology, University of Saskatchewan, Saskatoon, SK S7N 5A2, Canada; 4 Cardiovascular Division, Department of Medicine, University of Michigan, Ann Arbor, MI 48109, USA

**Keywords:** myocardial infarction, human, inflammation, clinical outcomes

## Abstract

Circulating monocytes have different subsets, including classical (CD14++CD16−), intermediate (CD14++CD16+), and nonclassical (CD14+CD16++), which play different roles in cardiovascular physiology and disease progression. The predictive value of each subset for adverse clinical outcomes in patients with coronary artery disease is not fully understood. We sought to evaluate the prognostic efficacy of each monocyte subset in patients with ST-elevation myocardial infarction (STEMI). We recruited 100 patients with STEMI who underwent primary percutaneous coronary intervention (PCI). Blood samples were collected at the time of presentation to the hospital (within 6 h from onset of symptoms, baseline (BL)) and then at 3, 6, 12, and 24 h after presentation. Monocytes were defined as CD45+/HLA-DR+ and then subdivided based on the expression of CD14, CD16, CCR2, CD11b, and CD42. The primary endpoint was a composite of all-cause death, hospitalization for heart failure, stent thrombosis, in-stent restenosis, and recurrent myocardial infarction. Univariate and multivariate Cox proportional hazards models, including baseline comorbidities, were performed. The mean age of our cohort was 58.9 years and 25% of our patients were females. Patients with high levels (above the median) of CD14+CD16++ monocytes showed an increased risk for the primary endpoint in comparison to patients with low levels; adjusted hazard ratio (aHR) for CD14+/CD16++ cells was 4.3 (95% confidence interval (95% CI) 1.2–14.8, *p* = 0.02), for CD14+/CD16++/CCR2+ cells was 3.82 (95% CI 1.06–13.7, *p* = 0.04), for CD14+/CD16++/CD42b+ cells was 3.37 (95% CI 1.07–10.6, *p* = 0.03), for CD14+/CD16++/CD11b+ was 5.17 (95% CI 1.4–18.0, *p* = 0.009), and for CD14+ HLA-DR+ was 7.5 (95% CI 2.0–28.5, *p* = 0.002). CD14++CD16−, CD14++CD16+, and their CD11b+, CCR2+, and CD42b+ aggregates were not significantly predictive for our composite endpoint. Our study shows that CD14+ CD16++ monocytes and their subsets expressing CCR2, CD42, and CD11b could be important predictors of clinical outcomes in patients with STEMI. Further studies with a larger sample size and different coronary artery disease phenotypes are needed to verify the findings.

## 1. Introduction

Cardiovascular diseases are the leading cause of mortality and morbidity worldwide [1]. ST-segment elevation myocardial infarction (STEMI) has the worst prognosis among cardiovascular diseases. STEMI is caused by the rupture of atherosclerotic plaque in the coronary arteries. The incidence of STEMI comprises around 40% of myocardial infarction (MI) presentations [2]. Despite significant advances in timely revascularization and medical therapy, 1-year mortality of STEMI can be as high as 20% [3]. Although the mortality rate for STEMI has significantly improved in the last decade, the risk of adverse clinical outcomes widely differs among various subgroups of STEMI patients [4]. Therefore, risk stratification of individual patients with STEMI is of paramount importance for individualized management strategies and allocating health care resources. Acute myocardial infarction (AMI) triggers a systemic and local inflammatory response, which initiates the mobilization and recruitment of a wide variety of inflammatory cells, including monocytes [5,6]. Monocytes have different subsets; classical (CD14++CD16−), intermediate (CD14++CD16+), and nonclassical (CD14+CD16++), which play different roles in cardiovascular physiology and cardiovascular disease progression [7]. CD14++CD16− monocytes play a significant role in triggering the inflammatory response after myocardial injury in STEMI and contribute to atherosclerosis [8]. Although previous clinical studies have shown that CD14++CD16− can predict adverse clinical outcomes in patients with coronary artery disease [8], other studies have shown that CD14++CD16− monocytes do not change after ischemic events, such as stroke [9]. CD14+CD16++ cells can have pro-inflammatory functions by secreting tumor necrosis alpha (TNF-α) and interleukin-1 (IL-1), which enhance the immune cell recruitment [10,11]. Moreover, they can have atheroprotective properties by enhancing efferocytosis [7]. Similarly, CD14++CD16+ monocytes have mixed functions and can be both pro-inflammatory and anti-inflammatory [9,12,13,14]. In this study, we provide a comprehensive temporal analysis of the dynamic changes in circulating innate immune cells, including monocytes and inflammatory markers, and their prognostic values in patients with STEMI.

## 2. Results

### 2.1. Baseline Characteristics

This prospective study included 100 patients with STEMI enrolled between August 2017 and July 2020. The baseline characteristics of the cohort are shown in Table 1. The study population was predominantly male (75%), and the mean age was 58.9 years (interquartile range-IQR 1.03). The median BMI of the study population was 28.8 (IQR 0.62), and the study population was predominantly white (92%). The right coronary artery was the culprit artery in 50% of patients, followed by the left anterior descending artery (31%) and the left circumflex artery (15%). In this study, 31% of our patients had DM, 67% had hypertension, 35% had hyperlipidemia, and 55% were current smokers. The mean follow-up of enrolled patients was 1.3 years.

### 2.2. Mobilization of Inflammatory Cells

**Figure 1:**
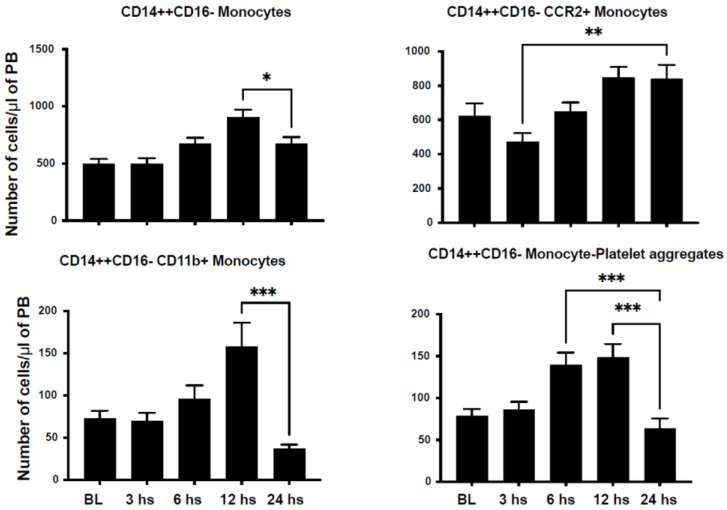
Classical monocytes expressing CD14++/CD16− are mobilized after myocardial infarction in humans. Bar graphs showing the number of circulating CD14++/CD16− monocytes in the peripheral blood after STEMI. The data demonstrate a peak of classical monocytes in the peripheral blood at 6 h after STEMI. The subset of classical monocytes expressing the activation markers CCR2, CD11b, and CD42 (monocyte–platelet aggregates) also peaked within 12–24 h after STEMI (data shown as mean ± SEM, * *p* < 0.05, ** *p* < 0.01, *** *p* < 0.001 compared to 24 h value).

**Figure 2:**
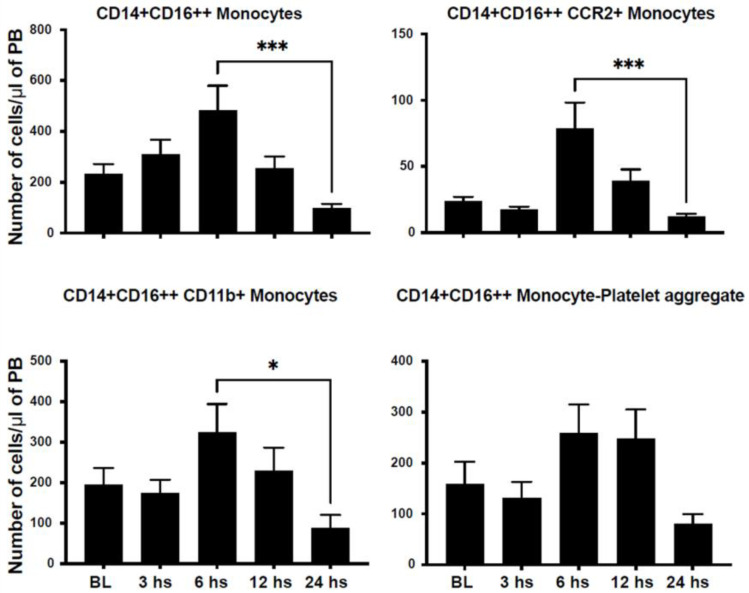
Non-classical monocytes expressing CD14+/CD16++ are mobilized after myocardial infarction in humans. Bar graphs showing the number of circulating CD14+/CD16++ monocytes in the peripheral blood after STEMI. The data demonstrate a peak of non-classical monocytes in the peripheral blood at 6 h after STEMI. The subset of non-classical monocytes expressing the activation markers CCR2, CD11b, and CD42 (monocyte–platelet aggregates) also peaked within 6–12 h after STEMI (data shown as mean ± SEM, * *p* < 0.05, *** *p* < 0.001 compared to 24 h value).

**Figure 3:**
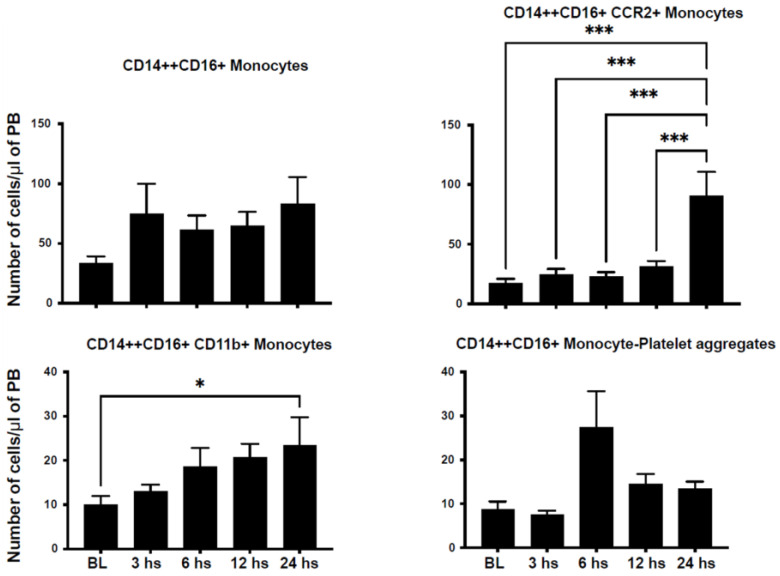
Intermediate monocytes expressing CD14++/CD16+ are mobilized after myocardial infarction in humans. Bar graphs showing the number of circulating CD14++/CD16+ monocytes in the peripheral blood after STEMI. The data demonstrate a bimodal peak of intermediate monocytes in the peripheral blood at 3 and 24 h after STEMI. The subset of intermediate monocytes expressing the activation markers CCR2 and CD11b also peaked within 24 h after STEMI. However, intermediate monocytes expressing CD42 (monocyte–platelet aggregates) peaked at 6 h after STEMI (data shown as mean ± SEM, * *p* < 0.05, *** *p* < 0.001 compared to 24 h value).

We assessed the number of circulating inflammatory cells in an attempt to characterize their dynamic mobilization after STEMI in humans. We characterized monocyte populations based on the expression of CD14 and CD16 into classical monocytes (CD14++CD16−), non-classical monocytes (CD14+CD16++), and intermediate monocytes (CD14++CD16+). CD14++CD16− monocytes peaked at 12 h and returned to nadir levels at 24 h after injury (Figure 1). Similarly, CD14+CD16++ monocytes peaked at 6 h after STEMI (Figure 2). However, CD14++CD16+ monocytes reached their peak 24 h after injury (Figure 3).

C-C Motif Chemokine Receptor 2 (CCR2) plays a critical role in immune cell chemotaxis to monocyte chemoattractant protein-1 (MCP-1), a chemokine that specifically mediates monocyte chemotaxis. MCP-1 is upregulated in tissue injury such as myocardial infarction and directs the mobilization of monocytes and their influx into the injured myocardium. We noticed a peak in CD14+CD16−/CCR2+ at 24 h after injury (Figure 1). However, we observed a dip in CD14+CD16++/CCR2+ counts at 3 h after injury, which then peaked at 6 h after injury (Figure 2). Similarly, CD14++CD16+/CCR2+ increased progressively to its maximum level at 24 h after STEMI (Figure 3). The expression of CD11b is a marker of immune cell activation/inflammation. CD14+CD16− monocytes expressing CD11b peaked at 12 h after injury and returned to nadir levels by 24 h after STEMI (Figure 2). The expression of CD11b on the surface of CD14+CD16++ peaked at 6 h (Figure 2) but at 24 h on CD14++CD16+ monocytes (Figure 3). Platelet–leukocyte aggregates are indicative of monocyte activation and have been linked to adverse clinical events. Our data indicate that platelet–monocyte aggregates peaked early after STEMI among most monocyte subpopulations. This is consistent with the early peak inflammatory response as represented by the cytokine data.

### 2.3. Changes in Plasma Cytokines after STEMI in Humans

**Figure 4:**
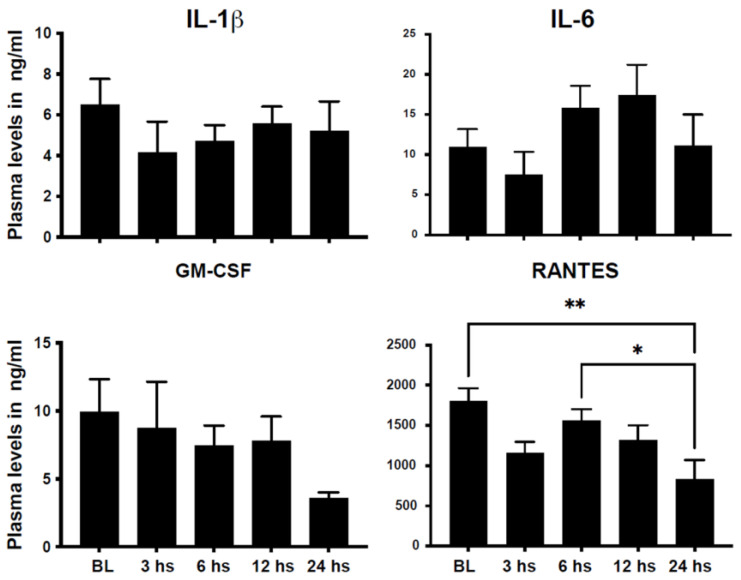
Plasma levels of pro-inflammatory cytokines show dynamic changes after STEMI. Bar graphs showing the plasma levels of the pro-inflammatory cytokines: interleukin-1 beta, interleukin-6, granulocyte-macrophage colony-stimulating factor, and RANTES (Regulated upon Activation, Normal T Cell Expressed and Presumably Secreted) cytokines. These data show dynamic changes in the plasma level with an early peak that precedes the mobilization of activated monocytes (data shown as mean ± SEM, * *p* < 0.05, ** *p* < 0.01, compared to 24 h value).

Chemokines and cytokines play an important role in the communication between injured tissue and hematopoietic cells and their progenitors in the bone marrow and spleen to regulate immune cell production and their mobilization to areas of need. We have recently shown that PB levels of markers of neutrophil activation such as myeloperoxidase (MPO) and S100A8/A9 are upregulated early after STEMI [15,16]. We assessed the plasma levels of classical chemokines/cytokines involved in myelopoiesis and monocytosis, such as IL-1β, IL-6, GM-CSF, and RANTES. Plasma levels of IL-1β, GM-CSF, and RANTES peaked early after STEMI, while the level of IL-6 peaked within 6–12 h after acute injury (Figure 4).

### 2.4. Correlation between Circulating Monocytes and Clinical Outcomes

**Figure 5:**
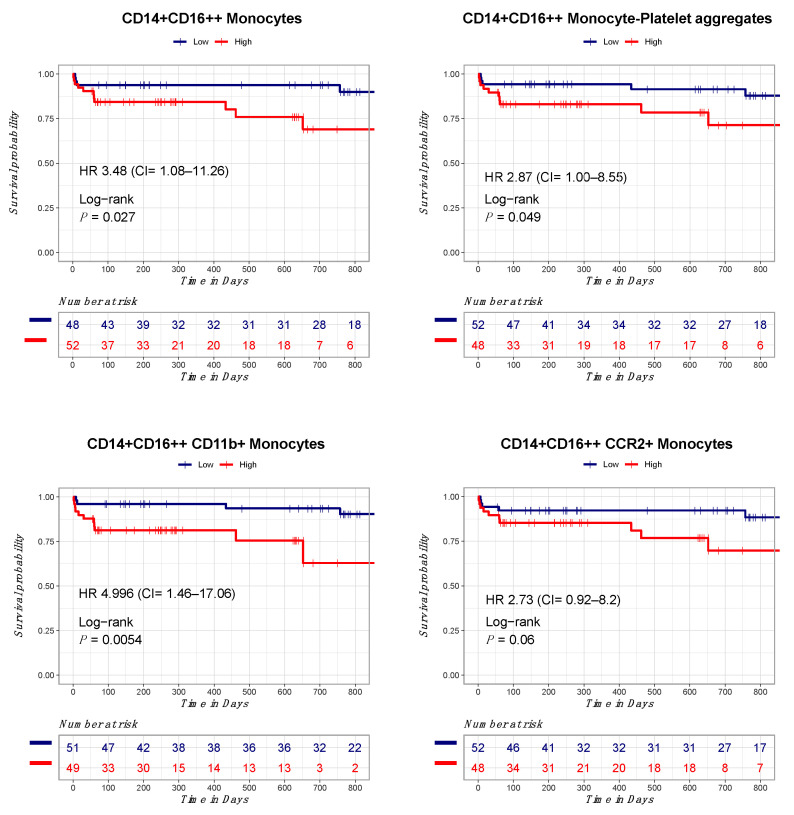
Elevated numbers of CD14+/CD16++ monocytes correlate with long-term adverse clinical events in STEMI patients. Survival curves for the probability of developing all-cause mortality, heart failure hospitalization, recurrent myocardial infarction, and stent thrombosis. These curves show a strong correlation between elevated numbers of circulating non-classical monocytes and their subsets expressing CD11b, CCR2, and their aggregates with platelets, and clinical events during long-term follow-up in STEMI patients.

We assessed the relationship between the number of circulating inflammatory cells, inflammatory cytokines, and clinical outcomes in our patient cohort. Patients were followed up for a median of 470 days. Overall, we observed 17 major adverse cardiac events during the follow-up period. Most of these clinical events were in the form of unplanned recurrent revascularization. Patients were divided based on the median number of circulating inflammatory cells at their peak into high level (above median) and low level (below median). In the univariate cox proportional hazards model, among all baseline characteristics, only age (HR = 1.06, 95% CI 1.01–1.11, *p* = 0.01), congestive heart failure (HR = 10.22, 95% CI 1.29–80.68, *p* = 0.02), and stroke (HR = 9.20, 95% CI 1.94–43.43, *p* = 0.005) significantly predicted the composite endpoint (Supplemental Table S1). In the univariate cox proportional hazards model, only total monocytes (CD14+/HLA-DR+) (3.80, 95% CI 1.19–12.13, *p* = 0.02), CD14+CD16++ monocytes (3.48, 95% CI 1.08–11.26, *p* = 0.03), and CD14+CD16++ monocytes expressing CD11b (4.99, 95% CI 1.46–7.06, *p* = 0.01) significantly predicted the clinical composite endpoint (Figure 5). Other monocyte populations such as CD14+CD16++/CD42b+ monocytes (2.86, 95% CI 0.95–8.55, *p* = 0.05) and CD14+CD16++/CCR2+ monocytes (2.73, 95% CI 0.91–8.11, *p* = 0.07) showed borderline significance towards predicting our composite endpoint (Supplemental Table S2). A close observation of the survival curves shows an early separation of the curves for all outcomes measured.

In the multivariate cox proportional hazards model, after adjusting for age, stroke, and congestive heart failure, CD14+CD16++ monocytes (adjusted HR (aHR) = 4.30, 95% CI 1.25–14.81, *p* = 0.02), CD14+CD16++/CCR2+ monocytes (aHR = 3.82, 95% CI = 1.06–13.76, *p* = 0.04), CD14+CD16++/CD42b+ monocytes (aHR = 3.37, 95% CI = 1.07–10.64, *p* = 0.03), and CD14+CD16++/CD11b+ monocytes (aHR = 5.17, 95% CI = 1.48–18.06, *p* = 0.009) remained significantly predictive of the clinical composite endpoint (Table 2).

## 3. Discussion

Myocardial infarction and the resulting heart failure are leading causes of morbidity and mortality worldwide. Several risk prediction models are available for risk stratification after STEMI, including the TIMI risk score and GRACE risk model. Recent studies as well as clinical guidelines have demonstrated the value of risk stratification for personalized medical care in the setting of myocardial infarction [17,18,19]. However, risk stratification models that incorporate biological variables, such as the immune response and markers of inflammation, are scarce. Changes in the epidemiological characteristics of MI and the availability of new biomarkers warrant an assessment of the performance of these scores in contemporary practice. We propose the addition of inflammatory and immune parameters to clinical predictive models to enhance their accuracy. Post-myocardial infarction inflammation is a major risk factor for adverse cardiac remodeling, heart failure, and major adverse cardiac events in short- and long-term follow-up. In this study, we systematically characterize innate immune cell mobilization and inflammatory cytokines in patients presenting with STEMI. Our data demonstrate dynamic changes in the number of circulating monocytes and their subsets as well as plasma cytokine levels. Peak numbers of monocytes followed cytokine levels suggesting a well-orchestrated immune response. The peak number of circulating monocytes and their subsets significantly predicted clinical outcomes in STEMI patients. Our results provide justification for incorporating inflammatory immune parameters in clinical risk stratification models. 

Myocardial injury triggers a series of signaling events to communicate with peripheral blood cells (PBCs) and bone marrow (BM) through processes that are just now being elucidated [20]. Monocytosis after AMI is a poor prognostic indicator, in part because monocytes may contribute to infarct expansion and impair cardiac remodeling, thereby promoting the progression to HF [21,22]. In the first wave of PBC response to AMI, classical (CD14++CD16−) monocytes and neutrophils aid in the clean-up after tissue damage through phagocytosis and the release of proteolytic enzymes. However, this initial injury response may actually confer long-term harm because the reduction in the initial recruitment of monocytes can reduce infarct size and prevent cardiac remodeling after AMI [23]. In addition to effects on the myocardium, monocytosis following AMI accelerates experimental atherosclerosis in animal models, thus initiating a vicious cycle; indeed, this type of cycle may contribute to recurrent coronary events in humans [24]. Our comprehensive study provides additional information regarding the prognostic value of specific monocyte subsets after STEMI. While the CD14++/CD16− cells have been traditionally described as the pro-inflammatory subset associated with adverse clinical events, our study suggests that other monocyte populations such as CD14+/CD16++ monocytes and their subsets were associated with adverse clinical events [8,13,21]. This can be explained by our specific monocyte markers that further defined certain populations of activated monocytes that express CD11b, CCR2, and the platelet marker CD42, which delineates platelet–monocyte complexes, a strong predictor of clinical events in coronary artery disease patients. We also examined the dynamics of circulating monocyte populations while other studies focused on one timepoint. These differences could explain the discrepancy between our results and others and provide additional markers for clinical risk stratifying in STEMI patients. Overall, we noticed that most of the monocyte populations tested peak within 12–24 h after STEMI and therefore, these timepoints represent the optimal timepoint in the clinical setting.

Circulating monocyte infiltration into the heart after myocardial infarction is largely considered a maladaptive response to sterile injury leading to scar expansion and cardiac dysfunction. Circulating monocytes are a heterogeneous population of immune cells that play a role in inflammation and tissue repair. In patients with STEMI, monocyte subsets have been associated with different aspects of prognosis [8,25,26,27,28,29]. Elevated monocyte levels upon hospital admission have been associated with adverse clinical outcomes in STEMI patients, such as recurrent infarction, heart failure, and increased mortality [28,30]. Furthermore, specific monocyte subsets have demonstrated differential prognostic implications in the context of STEMI. The pro-inflammatory CD14++CD16+ monocyte subset, in particular, has been linked to higher rates of cardiovascular events and poor clinical outcomes [13]. Additionally, a heightened CD14++CD16− monocyte count has been shown to independently predict future cardiovascular events [8]. These findings underscore the potential of circulating monocyte levels as a valuable prognostic tool in STEMI patients. In recent studies, monocyte–platelet aggregates have been found to correlate with poor outcomes in patients with acute myocardial infarction, suggesting a possible synergistic role between monocytes and platelets in driving the inflammatory response post-infarction [31,32]. This interaction is hypothesized to contribute to the initiation and propagation of inflammation and thrombosis, further aggravating the inflammatory response in the myocardium. Consequently, monitoring circulating monocyte levels and their subtypes could improve risk stratification and help tailor personalized therapeutic strategies for STEMI patients, potentially reducing the burden of adverse outcomes in this population. Our findings corroborate the available literature and provide additional insights into the monocyte subsets and their dynamic changes after STEMI and in relation to circulating inflammatory cytokines. Our study suggests that CD14+/CD16++ monocytes that express activation markers such as CD11b and CCR2 peak late after STEMI and are associated with adverse clinical events in STEMI patients. 

Our study highlights the prognostic value of circulating immune cells and inflammatory markers in STEMI patients. However, our study has multiple limitations. Given our cohort’s relatively small number, it is impossible to directly compare the prognostic value of circulatory monocytes with other models verified in large datasets, such as the TIMI and GRACE scores. Hence, future large cohort studies powered to examine these prognostic scores, either independently or in combination, are warranted. We conducted a stringent statistical analysis to account for the confounding factors on measured outcomes; however, there may be unmeasured or uncontrolled confounding factors that could influence the relationship between monocyte subsets and clinical outcomes in patients with STEMI that have not been assessed in our cohort. The study enrolled patients with revascularized STEMI; hence, the results may not be generalizable to other patient populations such as those with other forms of myocardial injury such as non-ST elevation myocardial infarction or patients undergoing elective percutaneous coronary interventions. Along the same lines, patients with non-revascularized STEMI, a rare occurrence in contemporary clinical practice, may have different patterns of circulating immune cells and inflammation, and accordingly, different outcomes.

In conclusion, our study demonstrates that circulating activated monocytes are associated with adverse clinical outcomes in patients with STEMI. This data provide evidence that incorporating biological markers of inflammation can enhance risk stratification models for STEMI patients and guide physicians to allocate more aggressive therapies to patients who need them the most. Future large, randomized studies utilizing biological parameters such as monocyte subsets are needed for risk stratification, the development of novel therapies, and long-term coronary artery disease monitoring.

## 4. Materials and Methods

### 4.1. Patient Enrollment

The study population consists of 100 patients with acute STEMI enrolled at the University of Kentucky hospitals between August 2017 and July 2020. STEMI was diagnosed based on EKG findings of new (or increased) and persistent ST-segment elevation in at least two contiguous leads of ≥1 mm in all leads other than leads V2–V3 where the following cut-off points apply: ≥2.5 mm in men <40 years old ≥2 mm in men >40 years old. Samples were collected at the time of presentation to the hospital (0 h; within 6 h from onset of symptoms; baseline (BL)) and then at 3, 6, 12, and 24 h after presentation. Five matched controls with similar comorbidities, but no active myocardial ischemia/infarction, were included in the analysis. Supposing momentarily that there is a single timepoint with a significance level of 5%, we estimate that a sample size of 67 patients will provide 80% or better power to detect an expected 15% change in circulating monocyte counts after STEMI based on published reports. The study protocol complies with the Declaration of Helsinki and was approved by the University of Kentucky’s Institutional Review Board and Ethics Committees [33]. All patients provided written informed consent at enrollment. Our primary outcome was a clinical composite endpoint of all-cause death (death from any cause as assessed by examining the hospital medical records and national social security death index), hospitalization for heart failure (hospital admissions with heart failure as the primary discharge diagnosis), stent thrombosis (recurrent myocardial infraction with confirmation of probable or definite stent thrombosis in the discharge summary), in-stent restenosis (confirmed stent restenosis on a coronary angiogram), and recurrent myocardial infarction (defined as rehospitalization of myocardial infarction that is diagnosed based on the STEMI EKG criteria detailed above or a significant rise and fall in cardiac biomarkers which is more than 5-fold the upper level of normal in the setting of symptoms and clinical criteria of myocardial injury). The clinical outcomes were assessed by a member of the research team (M.A-A.) and adjudicated by the senior author (A.AL.)

### 4.2. Flow Cytometry

For human peripheral blood inflammatory monocytes’ quantification, peripheral blood (PB) samples were stained against CD14 PE (Biolegend, San Diego, CA, USA), CD16 FITC (Biolegend, San Diego, CA, USA), HLA DR APC/Cy7 (Biolegend, San Diego, CA, USA), CD 42b PE/Cy7 (Biolegend, San Diego, CA, USA), CCR2 PerCP/Cy5.5 (Biolegend, San Diego, CA, USA), and CD11b APC (Biolegend, San Diego, CA, USA). Monocytes were defined as CD45+/HLA-DR+ and then subdivided based on the expression of CD14 and CD16 into classical (CD14++/CD16−), intermediate (CD14++/CD16+), and non-classical (CD14+/CD16++) (Supplemental Figure S1). Monocyte subpopulations were then classified based on their expression of CCR2, CD11b, and CD42. All samples were stained after lysis of the red blood cells and staining buffers included FC blocking to reduce nonspecific staining. Samples were acquired using an LSR II (Becton Dickinson, Mountainview, CA, USA) system and analyzed using FlowJo (version 7) software to generate dot plots and analyze the data.

### 4.3. Luminex Assay

At the pre-defined timepoints, plasma was collected using the PB collection protocol detailed earlier. Inflammatory biomarkers, such as interleukin-1 beta (IL-1β), IL-6, granulocyte-macrophage colony-stimulating factor (GM-CSF), and Chemokine ligand 5, known as Regulated upon Activation, Normal T Cell Expressed and Presumably Secreted (RANTES), were quantified using the Milliplex cytokine magnetic kit (MILLIPLEX MAP for Luminex xMap Technology, Millipore, Burlington, MA, USA) according to the manufacturer’s protocol.

### 4.4. Statistical Analysis

Baseline demographic characteristics were presented as mean with standard error (SE), median with interquartile range, or frequencies with percentages as appropriate. Patients were divided based on the median number of monocyte subpopulations. Patients who were above the median were considered the high-level group, and patients who were below the median were considered the low-level group. A univariate Cox proportional hazards model was performed to estimate the hazard ratio of our composite clinical endpoint in the high- compared to low-level groups for these parameters. Another univariate Cox proportional hazards model, including age, sex, body mass index, diabetes mellitus, hypertension, congestive heart failure, troponin-I, previous MI, stroke, chronic kidney disease, and peripheral vascular disease, was performed to estimate the hazards ratio of the same composite endpoint. Inflammatory cells that were significantly predictable to the composite endpoint in the univariate Cox proportional hazards model were entered in a multivariate Cox proportional hazards model with the baseline characteristic variables that were significantly predictable to the composite endpoint in the univariate Cox proportional hazards model. The dynamics of inflammatory cells over time were compared using repeated measure ANOVA or the Friedman test as appropriate. Because the hospital changed the troponin assay in the middle of the study, the numerical values of troponin-1 ranged from single digits to four digits. To overcome this irregularity in the data, we divided troponin-1 in each assay separately into quartiles, and each quartile received the same code in both assays. Hence, troponin-1 was analyzed as an ordinal variable rather than a numerical variable in our study. All statistical analyses were performed using R studio, version 1.4.1106 (R Foundation for Statistical Computing) and IBM SPSS Statistics, version 28.0, Armonk, NY, USA: IBM Corp.

## Figures and Tables

**Table 1:** Baseline characteristics of study population.

Demographic and Clinical Variables	N (%), Mean (SD), or Median (IQR)
Age	58.9 (1.03)
Sex, female	25 (25%)
BMI	28.8 (0.62)
Diabetes mellitus	31 (31%)
Hypertension	67 (67%)
Hyperlipidaemia	35 (35%)
Current smoker	55 (55%)
Congestive heart failure	1 (1%)
Baseline LVEF	46.07 (13.8)
Previous myocardial infarction	14 (14%)
Previous coronary revascularization	24 (24%)
Previous coronary bypass surgery	1 (1%)
Previous stroke	4 (4%)
Peripheral vascular disease	2 (2%)
Chronic kidney disease	1 (1%)
Door to balloon time in minutes *	33.5 (28.4)
Race	
White	92 (92%)
African American	5 (5%)
Culprit artery	
Left anterior descending	31 (31%)
Left circumflex	15 (15%)
Right coronary artery	50 (50%)
Medications at discharge	
Statins	97 (97%)
Angiotensin converting enzyme	63 (63%)
Angiotensin receptor blocker	10 (10%)
Beta blocker	93 (93%)
Aspirin	96 (96%)
P2Y12 inhibitors	100 (100%)
Ticagrelor	85 (85%)
Clopidogrel	11 (11%)
Prasugrel	4 (4%)

BMI, body mass index; LVEF, left ventricular ejection fraction. * Door to balloon time was available in only 41 patients.

**Table 2:** Multivariate cox proportional hazards model for a composite endpoint of all-cause death, myocardial infarction, recurrent hospitalization for heart failure, stent thrombosis, or in-stent restenosis.

Models	Hazardous Ratio	95% Confidence Interval	*p* Value
Model 1			
CD14+CD16++ monocytes	4.30	1.25–14.81	0.02
Age	1.06	1.01–1.11	0.007
Stroke	14.03	2.61–75.31	0.002
Congestive heart failure	18.41	1.92–176.40	0.01
Model 2			
CD14+CD16++/CCR2+	3.82	1.06–13.76	0.04
Age	1.05	1.01–1.11	0.01
Stroke	17.89	2.77–115.24	0.002
Congestive heart failure	16.52	1.69–161.28	0.01
Model 3			
CD14+CD16++/CD42b+	3.37	1.07–10.64	0.03
Age	1.06	1.01–1.11	0.007
Stroke	12.02	2.29–63.12	0.003
Congestive heart failure	18.04	1.87–174.13	0.01
Model 4			
CD14+CD16++/CD11b+	5.17	1.48–18.06	0.009
Age	1.05	1.01–1.10	0.01
Stroke	14.58	2.76–76.94	0.001
Congestive heart failure	15.64	1.62–150.95	0.01
Model 5			
CD14+ HLA-DR+	7.57	2.00–28.57	0.002
Age	1.09	1.03–1.16	0.001
Stroke	14.86	2.68–82.30	0.001
Congestive heart failure	23.59	2.38–233.17	0.006

## Data Availability

All data included in this paper are available upon reasonable request to the corresponding author.

## Supplementary Materials

The following supporting information can be downloaded at: https://www.mdpi.com/article/10.3390/ijms241411342/s1.
