# Supplementary material for: Prognostic Significance of Activated Monocytes in Patients with ST-Elevation Myocardial Infarction
Source: Int J Mol Sci. 2023 Jul 12;24(14):11342. doi: 10.3390/ijms241411342 (PMC10378894; doi:10.3390/ijms241411342)
Supplement: Supplementary file 1 [file ijms-24-11342-s001.zip › ijms-2497179-supplementary.pdf]

Table S1. Univariate analysis Cox Proportional-Hazards Model of baseline characteristics for a composite endpoint of all-cause death, myocardial infarction, recurrent hospitalization for heart failure, stent thrombosis, or in-stent restenosis

Variable	HR	95%CI	P value
**Age**	1.06	1.01 - 1.11	0.01
**Sex**	0.64	0.18 - 2.34	0.52
**Body Mass index**	0.92	0.83 - 1.01	0.09
**Diabetes Mellitus**	0.74	0.23 - 2.34	0.61
**Hypertension**	1.27	0.40 - 3.99	0.67
**Congestive Heart Failure**	10.22	1.29 - 80.68	0.02
**Troponin-1**	1.18	0.71 - 1.97	0.51
**Previous MI**	3.03	0.94 - 9.75	0.06
**Stroke**	9.20	1.94 - 43.43	0.005
**Chronic Kidney Disease**	1.08	0 - Inf	0.99
**Peripheral vascular disease**	3.98	0.51 - 30.71	0.18

HR, hazardous ratio; CI, confidence interval; MI, myocardial infarction.

Table S2. Univariate analysis Cox Proportional-Hazards Model of inflammatory cells for a composite endpoint of all-cause death, myocardial infarction, recurrent hospitalization for heart failure, stent thrombosis, or in-stent restenosis

Variable	HR	95%CI	P value
**CD14+/HLA-DR+**	3.80	1.19 - 12.13	0.02
**CD14+CD16– Monocytes**	0.94	0.32 - 2.71	0.91
**CD14+CD16–/CCR2+**	0.67	0.22 - 2.00	0.47
**CD14+CD16–/CD11b+**	1.94	0.65- 5.79	0.23
**CD14+CD16–/CD42b+**	1.44	0.51 - 4.03	0.48
**CD14++CD16+ Monocytes**	1.28	0.45 - 3.68	0.63
**CD14++CD16+/ CCR2+**	1.24	0.44 - 3.48	0.68
**CD14++CD16+/ CD11b+**	0.62	0.20 - 1.84	0.39
**CD14++CD16+/ CD42b+**	1.61	0.57 - 4.54	0.36
**CD14+CD16++ Monocytes**	3.48	1.08 - 11.26	0.03
**CD14+CD16++/ CCR2+**	2.73	0.91 - 8.11	0.07
**CD14+CD16++/ CD11b+**	4.99	1.46 - 7.06	0.01
**CD14+CD16++/ CD42b+**	2.86	0.95 - 8.55	0.05

HR, hazardous ratio; CI, confidence interval.

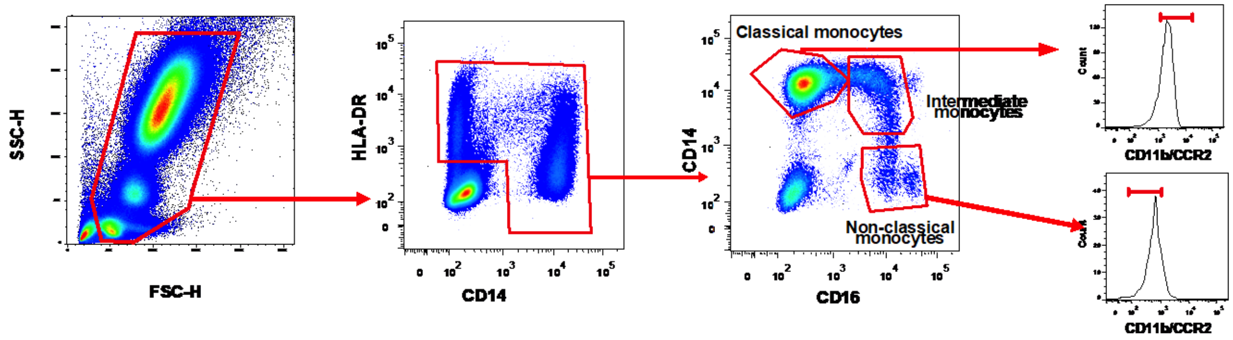

Figure S1. Flow cytometry gating strategy demonstrating the gating method for monocytes and their classification into classical, intermediate, and non-classical monocytes.
